# Political Ideology and Stigmatizing Attitudes Toward Depression: The Swedish Case

**DOI:** 10.15171/ijhpm.2019.15

**Published:** 2019-03-19

**Authors:** Jesper Löve, Monica Bertilsson, Johan Martinsson, Lena Wängnerud, Gunnel Hensing

**Affiliations:** ^1^ Department of Public Health and Community Medicine at Institute of Medicine, University of Gothenburg, Gothenburg, Sweden.; ^2^ Department of Political Science, University of Gothenburg, Gothenburg, Sweden.

**Keywords:** Political Ideology, Depression, Stigmatizing Attitudes, Mental Health, Sweden

## Abstract

**Background:** Stigmatizing attitudes toward persons with mental disorders is a well-established and global phenomenon often leading to discrimination and social exclusion. Although previous research in the United States showed that conservative ideology has been related to stigmatizing attitudes toward mental disorders, there is reason to believe that this mechanism plays a different role in the context of a universal welfare state with a multi-party system such as Sweden. Furthermore, "mental disorders" may signify severe psychotic disorders, which may evoke more negative attitudes. This suggests the importance of specific studies focusing on the more common phenomenon of depression. This paper investigates the relationship between political ideology and stigmatizing attitudes toward depression in Sweden.

**Methods:** This study is part of the New Ways research program. Data were collected by the Laboratory of Opinion Research (LORE) at the University of Gothenburg in 2014 (N = 3246). Independent variables were political ideology and party affiliation. The dependent variable was the Depression Stigma Scale (DSS). Data were analyzed with linear regression analyses and analyses of variance.

**Results:** More conservative ideology (B = 0.68, standard error [SE] = 0.04, *P*<.001) and more conservative party affiliation (F(8 2920) = 38.45, *P*<.001) showed more stigmatizing attitudes toward depression. Item-level analyses revealed a difference where the supporters of the conservative party differed (*P*<.05) from supporters of the liberal party, with a higher proportion agreeing that "people could snap out of " depression if they wanted to; the populist right-wing party differed from the conservative party with a higher proportion agreeing on items displaying people with depression as "dangerous" and "unpredictable." Even self-stigma was highest among the populist right-wing party with 22.3% agreeing that "if I had depression I wouldn’t tell…."

**Conclusion:** Political ideology was associated with stigmatizing attitudes toward depression in Sweden. The results also confirm the need to distinguish between different forms of conservatism by observing social distance as being a more important driver among voters for the populist right-wing party compared with personal agency and responsibility among voters for the more traditional conservative party.

## Background

Stigmatizing attitudes toward people with mental disorders are a well-established and global phenomenon often leading to discrimination and social exclusion.^1^ There are several reasons why stigma toward mental disorders occurs at micro, meso, and macro levels.^2^ A number of studies suggest that political ideology is an important part of the dynamic.^3-5^ However, a recent literature review found that population-based studies of attitudes toward depression in relation to political ideology are a neglected topic in favor of studies of the more generic “mental disorders.”^6^ Results, mostly from research in the United States, indicate that political conservatism is associated with stigma around mental disorders; people to the right of the ideological spectrum tend, more than others, to agree “that a person with mental illness is a danger to others” and decline “to live nearby someone with a mental health problem.”^3^ Thus, the impact of political ideology appears in analysis of negative stereotypes, an attitudinal component of stigma, as well as in analysis of social distance, a behavioral component. The overall aim of this study was to move research forward regarding the relationship between political ideology and stigmatizing attitudes toward depression. Considering the interdisciplinary nature of this issue, the present study was a collaboration between public health researchers and political scientists, in line with a previous call from Gagnon et al^7^ in the *International Journal of Health Policy and Management*. To fulfil our overall aim, we make 2 main contributions to the literature.

First, we build on recent research^8,9^ highlighting that sole reliance on right-wing authoritarianism (RWA) theories contributes to an oversimplification of complex relationships; political ideology in general and conservatism in particular are multi-dimensional phenomena. This is also in line with a recent study by DeLuca et al^10^ emphasizing the need for studies investigating the theoretical underpinnings of the relationship between RWA and mental health stigma. Feldman and Johnston^9^ suggest that conservatives, compared with liberals, may score higher on authoritarianism, but at the same time there are reasons to expect that individuals who score high on economic conservatism are different from other conservatives because links between authoritarianism and economic conservatism are weak. From a political ideology perspective, it is therefore important to examine to what extent there are significant differences between liberals and conservatives on the one hand and among different groups of conservatives on the other hand. The multi-party system in Sweden makes this possible.

Second, we studied stigmatizing attitudes toward depression rather than the broader concept of mental disorders. Mental disorders may signify severe psychotic disorders in the minds of respondents, which might evoke negative stereotypes in a way that is not illustrative of people’s attitudes to more common mental disorders. Depression affects 300 million people globally and is a leading cause of disability throughout the world.^11^ In Sweden, the lifetime risk of depression has been estimated to be 30%–40% in women and 20% in men.^12^ From a public health perspective, it is important to know whether the relationship between political ideology and stigmatizing attitudes also occurs in relation to depression.

Previous research shows that adverse public attitudes toward depression may result in discrimination. First, stigmatizing attitudes may impose individual or inter-relational discrimination, given that people with mental illness may be avoided, treated badly, or looked down upon.^13^ Second, people with mental illness may also embrace the negative label that has been put on them.^14^ Such self-stigma may result not only in cognitive concerns such as bad self-esteem but also in adverse health-related behavior, for example, delay in seeking help^15^ and enhanced self-imposed social isolation.^16^

Stigmatizing attitudes have been explained as an individual response to a psychologic need. “The closed authoritarian personality syndrome” has been suggested as the core mechanism linking conservatism to stigma.^17^ However, recent studies have criticized the strong focus on personality as the root of political orientation, suggesting a synthesis with contextual factors. Socio-psychologic explanations stress how stigma is “defined in and enacted through social interaction”^2^ in the same way that people’s political orientation is shaped in interactions with the environment.^18^ The importance of social interaction and previous life experiences has been indicated in previous research observing that correlates such as female gender, higher education, younger age, and previous contact with individuals with mental illness are associated with less stigmatizing attitudes.^3,19^ Even though stigma toward mental disorders is a phenomenon observed globally, the national environment may provide variations in how and to what extent stigma is manifested.

To the best of our knowledge, this is the first study on whether there is a link between political ideology and stigmatizing attitudes toward depression. Moreover, it is novel to study this link in a country such as Sweden, characterized by a universal welfare state, which, according to previous research, should lead to lower levels of stigmatizing attitudes in such relationships. Even more important is that Sweden has a multi-party system with various types of conservative parties ranging from traditional conservative parties, focusing on economic issues and family values, to populist right-wing parties, focusing on issues of anti-immigration and law and order.^20^ One indication of the need for more nuanced understandings of the association with conservatism is the finding in previous research that supporters of different conservative parties in Sweden vary significantly in levels of anxiety. People supporting the Moderate Party, a traditional conservative party, are consistently found among the least anxious in Sweden with regard to both personal risks and threats, such as being a victim of crime, and to social risks and threats, such as impaired welfare. This low level of anxiety is hard to reconcile with the assumption of stigma as a psychologic process for managing fear and uncertainty. In contrast, people supporting the Sweden Democrats, a populist right-wing party, are among the most anxious in Sweden.^21^

In order to address our aim, we posed the following 2 hypotheses:

H1. Conservative political ideology is linked to stigmatizing attitudes toward depression in a universal welfare state such as Sweden.

H2. There are significant differences between supporters of the Moderate Party versus the Sweden Democrats, where attitudes linked to personal agency and responsibility are prominent among Moderate Party supporters and values linked to predictability and fear are prominent among supporters of the Sweden Democrats.

## Methods

### Study Sample

This study is part of the New Ways research program at Sahlgrenska Academy, University of Gothenburg, with the overall objective to support work participation in persons with common mental disorders. The data were collected by the Laboratory of Opinion Research (LORE) at the University of Gothenburg, through its Citizen Panel. The Swedish Citizen Panel is a web-based panel where people regularly participate in online surveys on various topics. LORE utilizes different recruitment techniques, and for this study, respondents were from a self-recruited panel of the Swedish population. The fieldwork started on November 27, 2014 with an email invitation to participate in the study and closed on December 21, 2014. This was approximately 2 months after the regular national election in 2014. National elections are held every 4 years in Sweden. A total of 4840 individuals received the invitation. A maximum of 2 reminders were sent to those who did not respond. The study sample of 3246 individuals comprised almost equal proportions of women (47.3%) and men (52.7), with a slight imbalance toward older people (67.3% >45 years) and highly educated people (56.9% with a university degree; see Table 1). The Citizen Panel has been approved by the Regional Ethical Review Board in Gothenburg, Sweden (Dnr: 189-14).

### Measures

#### Dependent Variable: Index Measuring Stigmatizing Attitudes Toward Depression

The 9-item subscale Depression Stigma Scale (DSS)^22^ was used (personal subscale) as the dependent variable. The index was created as the sum of the nine statements with a 5-point (Likert) response scale ranging from “strongly disagree” (1) to “strongly agree” (5). Only individuals with a value for all items received a score for the final index (n = 3129), ranging from 9 to 45, with a higher score representing more stigmatizing attitudes.

#### Independent Variables: Political Ideology and Party Affiliation

Political ideology and party affiliation were the main independent variables. In line with previous studies on the effect of political ideology in Sweden, political ideology was captured by an index created from 4 statements that the respondents could either agree or disagree with on a 5-point Likert scale from “totally disagree” (1) to “totally agree” (5).^23^ The statements were preceded by the question “What do you think about the following propositions:” “Sweden should receive fewer refugees,” “The tax levels should be lower,” “The taxes on CO_2_ for petrol should be raised,” and “There should be a decrease of societal income inequalities.” The first and second statements were reversed in the final index. Party affiliation was measured by the following question: “What political party did you vote for in the national election in 2014?” (data collection was conducted 2 months after the national election in 2014). Response alternatives were all eight parties in the Swedish parliament (the Left Party, the Social Democrats, the Green Party, the Centre Party, the Liberal Party, the Moderate Party, the Christian Democrats, and the Sweden Democrats) and the largest party outside parliament (ie, Feminist Initiative).

The second set of analyses focused on H2. There are significant differences between supporters of the Moderate Party versus the Sweden Democrats in the kind of stigmatizing attitudes that emerge; values linked to personal agency are prominent among Moderate supporters and values linked to predictability and fear are prominent among supporters of the Sweden Democrats should party affiliation be seen as a proxy for underlying ideology. It is not self-evident how to distinguish between the Swedish parties, but because this study relied on a population-based sample, we departed from the Swedish National Election Study 2014 in which respondents were asked to classify the political parties in Sweden on an ideological left-right scale. The results showed that respondents viewed the Moderate Party and the Sweden Democrats as the most right-wing parties, whereas the Christian Democrats, the Liberal Party, and the Centre Party were viewed as center-right parties; the Green Party and the Social Democrats were viewed as center-left parties, and the Left Party and the Feminist Initiative as left-wing parties.^23^ Based on this, we categorized the Moderate Party and the Sweden Democrats as “conservatives.” For the sake of simplicity, we chose the Liberal Party to represent underlying liberal ideology (among the center-right parties, this is also the largest party in our sample).

We performed 2 sets of comparisons and examined whether there were significant differences (1) between liberals and conservatives and (2) between different groups of conservatives.

### Covariates

Because attitudes toward depression may be associated with gender, educational level, and age,^22^ these 4 measures were used in adjusted models. Education was divided into 5 categories: (1) elementary school or less, (2) upper secondary, (3) post-secondary but not university, (4) university, and (5) doctoral degree (PhD). The analyses were also adjusted for self-reported health measured with one question on general health, with response alternatives on a 5-point Likert scale ranging from “very good” to “very bad.”

### Statistical Analyses

Descriptive statistics for the study sample are presented as proportions (%) and frequencies (n). For the DSS and the political ideology index, the mean and standard deviation (SD) were calculated for the total indexes and for each item. To assess the relationship between political ideology (ie, independent variable) and stigmatizing attitudes (ie, dependent variable), linear regression analyses were conducted and the unstandardized regression coefficient (B), standard error (SE), and adjusted R^2^ were calculated. To investigate the relationship between party affiliation and the mean level of stigmatizing attitudes, analysis of variance (ANOVAs) and post hoc comparisons (Scheffe) were calculated with a statistical significance level of *P* < .001. Both the linear regression and the ANOVA analyses comprised of individuals with values on all items in the index alone, excluding 117 individuals or 3.6% of the total study sample. However, sensitivity analyses were conducted including all cases. To explore this relationship further, additional analyses were conducted between (1) individual items of the DSS and party affiliation by the use of repeated Kruskal-Wallis H tests and (2) individual items of the DSS and political ideology by the use of repeated ordinary least squares (OLS) regression analyses with *P* <.001. OLS regressions adjusting for gender, education, age, and self-reported health were also performed. To investigate proportional differences per item of the DSS (ie, proportion that agreed or strongly agreed) in relation to political affiliation, proportions and their differences were calculated with 95% CIs.^24^ All calculations were performed in Stata SE version 15.1.

## Results

### Sample Characteristics

The overall response rate within the sample from the panel was 67% (ie, 3246 of the 4840 who were invited). There was a higher proportion of people over 45 years and people with a university education (Table 1).

**Table 1:** Demographic Characteristics of the Study Sample

**Demographic Characteristics**	**Proportion (%)**	**Frequency (n)** ^a^
Gender		
Women	47.3	1494
Men	52.7	1666
Age		
15–30 years	9.2	290
31–45 years	22.5	712
46–60 years	31.0	987
60+ years	37.3	1182
Education		
Primary or less	5.4	170
Upper secondary	21.9	696
Post-secondary	12.4	394
University	56.9	1809
Doctoral degree	3.4	108

^a^Minor inconsistencies between the figures and total study sample are due to internal missing values.

### Descriptive Statistics Stigmatizing Attitudes Toward Depression Index: Total Sample

The mean score for stigmatizing attitudes toward depression in the total sample was 15.9 with SD of 5.5 and an acceptable internal consistency (Cronbach α = 0.78). Mean values were low overall, with indications of floor effects on some items, for example, “personal weakness” and “avoid people with depression” (Table 2).

**Table 2:** Items Included in the Index of Stigmatizing Attitudes Toward Depression: Summary Statistics

**Items**	**n**	**Mean (Range 1-5)** ^a^	**SD**
People with depression could snap out of it, if they wanted	3175	2.04	1.06
Depression is a sign of personal weakness	3178	1.38	0.78
Depression is not a real medical illness	3176	1.56	1.04
People with depression are dangerous	3180	1.60	0.89
It is best to avoid people with depression so you do not become depressed yourself	3177	1.31	0.69
People with depression are unpredictable	3179	2.09	1.07
If I had depression, I would not tell anyone	3169	2.00	1.13
I would not employ someone if I knew they had been depressed	3165	2.14	1.22
I would not vote for a politician if I knew they had been depressed	3166	1.82	1.12

Abbreviation: SD, standard deviation. ^a^Mean for each variable with response alternatives (1) “strongly agree” to (5) “strongly disagree.”

### Descriptive Statistics Index of Political Ideology

The index capturing political ideology shows an acceptable internal consistency; Cronbach α = 0.68 and means did not indicate any major skewness (Table 3).

**Table 3:** Items Included in the Index of Political Ideology: Summary Statistics

**Items** ^a^	**n**	**Mean**	**SD**
Sweden should receive fewer refugees (reversed in index)	3187	3.00	1.41
Lower the taxes (reversed in index)	3183	3.33	1.21
Increase CO_2_ taxes for petrol	3183	3.00	1.25
Decrease income inequalities in society	3185	2.21	1.14

Abbreviation: SD, standard deviation. ^a^The question used reads as follows: Below, you will find a number of suggestions that have occurred in the political debate. What is your opinion on these matters? (1) Receive fewer refugees in Sweden, (2) Lower the taxes, (3) Increase CO_2_ taxes for petrol, and (4) decrease income inequalities in society. Five-point response scale (Likert) from totally disagree (1) to totally agree (5).

### Descriptive Statistics on Party Affiliation in the 2014 National Election in Sweden

Results from the 2014 general election in Sweden are reported in Table 4 in relation to the distribution of party affiliation in our sample. The proportions of supporters for the Left Party, the Liberal Party, the Green Party, and Feminist Initiative were higher in our sample compared with the results from the election in 2014. The relationship between political ideology and party affiliation showed the expected pattern with lower means for voters for left-wing parties and higher means for voters for right-wing parties (Table 4).

**Table 4:** Proportions (%) and Frequencies (n) of Self-reported Party Affiliation at the 2014 General Election in Sweden in the Study Sample Compared with the Election Results, and Summary Statistics on Political Ideology in Relation to Party Affiliation

**Party affiliation**	**Self-reported Affiliation: Proportion of the Study Sample (%)**	**Frequency (n)**	**The Results from the Election 2014: Proportion (%)**	**Mean for Political Ideology Index (Range 4-20)** ^a^	**SD for the Political Ideology Index**
Feminist Initiative	5.6	177	3.1	9.5	1.8
Left Party	10.3	309	5.7	10.2	2.0
Social Democrats	20.4	648	31.0	11.4	2.1
Centre Party	5.8	184	6.1	11.7	2.3
Liberal Party	8.7	276	5.4	11.6	2.2
Moderate Party	20.3	644	23.3	12.5	2.1
Christian Democrats	4.6	146	4.6	12.4	2.3
Green Party	10.6	336	6.9	9.9	1.8
Sweden Democrats	8.6	272	12.9	13.8	2.1

Abbreviation: SD, standard deviation. ^a^Mean for the index on political ideology comprised 5 statements with response alternatives from totally disagree (1) to totally agree (5).

### The Link Between Political Ideology and Stigmatizing Attitudes Toward Depression

Testing the first hypothesis (ie, conservative political ideology was linked to stigmatizing attitudes toward depression also in a universal welfare state such as Sweden), linear regression analyses showed that a more right-wing ideology (measured through an index) was associated with a higher degree of stigmatizing attitudes toward depression (B = 0.68, SE = 0.04, *P* < .001, adjusted R^2^ = 0.09). The association remained, even when adjusting for gender, education, self-reported health, and age (B = 0.60, SE = 0.04, *P* < .001, adjusted R^2^ = 0.14) (Table 5). Sensitivity analyses including all cases (ie, including the 117 individuals who did not have full information on all items in the stigma index) showed similar results as in the main analysis (B = 0.68, SE = 0.04, *P* < .001). These results persisted even when adjusting for gender, education, self-reported health, and age (B = 0.60, SE = 0.04, *P* < .001).

**Table 5:** Linear Regression Analysis for Political Ideology and Stigmatizing Attitudes Toward Depression, Crude (Model 1) and Adjusted for Gender, Education, Self-reported Health, and Age (Model 2)

**Item**	**Model 1**		**Model 2**	
	**Coefficient**	**SE**	**Coefficient**	**SE**
Ideology	0.68	0.04^a^	0.60	0.04^a^
Gender			2.34	0.19^a^
Education			−0.43	0.09^a^
Self-reported health			0.01	0.11
Age			−0.03	0.001^a^

Abbreviation: SE, standard error. ^a^*P* < .001.

The results above refer to the index created from 4 items commonly used to capture left-right political ideology in Sweden. The results looked basically the same if we instead turn to the measure on party affiliation: an ANOVA showed statistically significant differences in means of the stigma index (*F*(8,3) = 38.45, *P* < .001, η^2^ = 0.095) in relation to party affiliation. Sensitivity analyses including all cases (ie, including the 117 individuals who did not have full information on all items in the stigma index) showed similar results as in the main analysis (*F*(8,3) = 36.46, *P *< .001). Taken together, these 2 analyses—one using an index of political ideology and the other a measure of party affiliation—demonstrated that in a multi-party welfare state such as Sweden, we find an association between political ideology and stigmatizing attitudes toward depression; people on the right of the ideological spectrum tended, more than others, to display stigmatizing attitudes. Statistically significant associations (*P *< .001) were found between party affiliation and all items in the stigma index (repeated Kruskal-Wallis H tests) and between political ideology and all items in the stigma index (*P *< .001) (repeated OLS regression analyses; details not shown).

### A Closer Look at Differences Between Political Parties

Our second hypothesis was tested in this section. Is it possible to distinguish between various forms of conservative ideology having an effect on stigmatizing attitudes in Sweden? Table 6 shows mean values for the stigma index among supporters of the nine largest political parties in Sweden.

**Table 6:** Mean Values and SDs for the Stigma Index (9–45) Among Supporters for the 9 Largest Political Parties in Sweden

**Party**	**Mean (Range 9-45)**	**SD**
Feminist Initiative	12.99	3.76
Left Party	13.43	4.00
Social Democrats	15.41	5.19
Green Party	14.65	4.65
Centre Party	15.44	4.52
Liberal Party	15.93	5.14
Moderate Party	17.50	5.85
Christian Democrats	16.56	5.42
Sweden Democrats	19.08	6.63

Abbreviation: SD, standard deviation.

The results in Table 6 confirm that political ideology matters for stigmatizing attitudes: the lowest values, the least stigmatizing attitudes, are found within the Feminist Initiative and the Left Party, which both are left-leaning parties. Mean values for supporters of the Social Democrats, the Green Party, the Centre Party, and the Liberal Party were higher than the mean values for the 2 leftist parties but lower than for the Moderate Party, the Christian Democrats, and the Sweden Democrats.

Supporters of all parties (except the Christian Democrats) displayed statistically significantly fewer stigmatizing attitudes than supporters of the traditional conservative party, the Moderate Party, as well as the Sweden Democrats. However, supporters of the Moderate Party were, in turn, significantly different from the Sweden Democrats (Table 7). To get a more in-depth explanation for the difference, additional analyses separating various items in the stigma index were performed.

**Table 7:** Post Hoc Comparison (Scheffe) of the Stigma Index and Party Affiliation

**Row Mean – Column Mean**	**Left**	**Social Democrats**	**Green**	**Centre**	**Liberal**	**Moderate**	**Christian Democrats**	**Sweden Democrats**
Social Democrats	**1.99**							
	**0**							
Green	1.22	−0.76						
	0.36	0.79						
Centre	**2.01**	0.03	0.79					
	**0.03**	1	0.95					
Liberal	**2.51**	0.52	1.28	0.50		‏		
	**0**	0.98	0.34	1				
Moderate	**4.08**	**2.09**	**2.85**	**2.06**	**1.57**			
	**0**	**0**	**0**	**0.01**	**0.03**			
Christian Democrats	**3.14**	1.15	1.92	1.12	0.63	−0.94		
	**0**	0.69	0.1	0.89	1	0.88		
Sweden Democrats	**5.65**	**3.67**	**4.43**	**3.64**	**3.15**	**1.58**	**2.52**	
	**0**	**0**	**0**	**0**	**0**	**0.03**	**0.01**	
Feminist Initiative	−0.44	−**2.42**	−1.66	−**2.45**	−**2.94**	−**4.51**	−**3.58**	−**6.09**
	1	**0**	0.17	**0.01**	**0**	**0**	**0**	**0**

Differences between mean on stigma index in percentage points (*P* values).

### Separating Various Stigma Index Items

Of the nine statements included in the stigma index, 2 items are clearly related to fear and uncertainty: “People with depression are dangerous” and “People with depression are unpredictable;” thus they can be seen as a sign of RWA. Other items, however, can be seen as more closely related to a dimension of personal agency (interpreted as being responsible and trustworthy): “People with depression could snap out of it, if they wanted,” “I would not hire a person if I knew they had been depressed,” and “I would not vote for a politician if I knew they had been depressed.” Table 8 shows the percentages of respondents answering “strongly agree” or “agree” (categories merged) on the nine different items by party affiliation. The Sweden Democrats displayed the highest levels (strongly agree and agree) on all nine items, with the highest figure, 28.7%, for the item “I would not hire a person if I knew they had been depressed” and the lowest figure, 3.7%, for the item “It is best to avoid people with depression….” Supporters of the Moderate Party displayed the second highest levels (strongly agree and agree) with one exception; Christian Democrats, Liberals, and supporters of the Centre Party displayed higher levels for the item “If I had depression I would not tell anyone.” There was also a difference between the supporters of the Moderate Party and the Liberal Party, and the same was true for the differences between the supporters of the Moderate Party and the Sweden Democrats.

**Table 8:** Proportional Relationship Between Party Affiliation and Items from the DSS: Percentage (%) who Strongly Agree or Agree

**DSS Item**	**Party Affiliation**								
	**Left**	**Social Democrats**	**Green**	**Centre**	**Liberal**	**Moderate**	**Christian Democrats**	**Sweden Democrats**	**Feminist Initiative**
People with depression could snap out of it if they wanted	4.2	9.0	8.1	9.8	8.4	16.1	10.2	18.0	2.3
Depression is a sign of personal weakness	2.0	2.9	1.8	1.1	3.7	4.4	4.2	7.8	0.6
Depression is not a real medical illness	7.2	6.8	5.1	5.4	7.7	9.5	6.9	12.0	3.4
People with depression are dangerous	1.6	4.6	2.4	3.8	4.0	4.1	3.5	11.2	3.4
It is best to avoid people with depression so you don’t become depressed yourself	0.7	2.6	1.8	0.5	1.1	2.7	0.7	3.7	1.1
People with depression are unpredictable	5.2	9.0	11.9	9.8	10.6	14.6	11.8	22.3	6.9
If I had depression I would not tell anyone	7.5	10.2	9.6	12.6	12.8	12.4	14.6	20.3	5.1
I would not hire a person if I knew they had been depressed	4.9	11.9	10.5	13.2	17.0	21.2	18.6	28.7	5.7
I would not vote for a politician if I knew they had been depressed	4.6	8.4	5.7	7.7	8.4	13.6	6.9	19.9	1.7

Abbreviation: DSS, Depression Stigma Scale.

**Figure 1:**
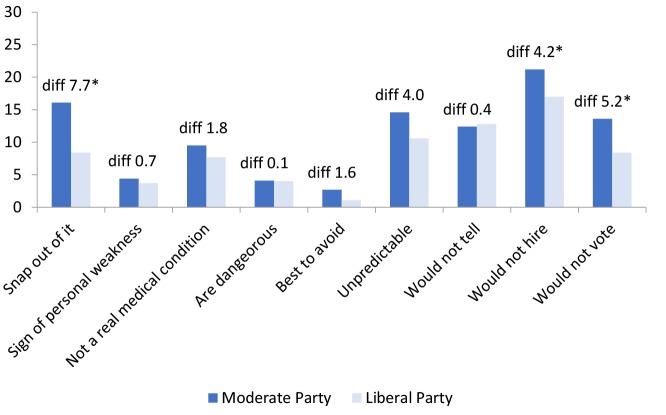
Comparing Supporters of the Moderate Party and the Liberal Party Across Stigma Index Items. Bars show percentages who strongly agree and agree (merged). Differences between groups in percentage points with statistically significant differences (*P* < .05) are marked with *.

**Figure 2:**
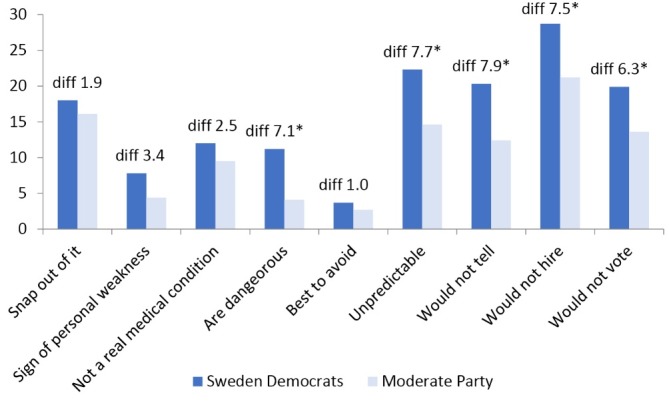
Comparing Supporters of the Sweden Democrats and the Moderate Party across stigma index items. Bars show percentages who strongly agree and agree (merged). Differences between groups in percentage points. * *P* < .05.

Figures 1 and 2 include 2 sets of comparisons between (1) answers among supporters of the Moderate Party versus supporters of the Liberal Party and (2) supporters of the Moderate Party versus supporters of the Sweden Democrats. The bars show the percentages for strongly agree and agree (categories merged), and the numbers show differences between groups on each item. An asterisk (*) indicates whether the difference is significant at a *P* value of .05 or lower. Figure 1 shows that there were statistically significant differences between the supporters of the Moderate Party versus the Liberal Party on 3 items: “People with depression could snap out of it if they wanted,” “I would not hire a person if I knew they had been depressed,” and “I would not vote for a politician if I knew they had been depressed.” The item “People with depression are dangerous” displayed the smallest difference (0.1 percentage point, non-significant) among all items included. This indicates that there is a difference between supporters for the Liberal Party and the Moderate Party in stigmatizing attitudes, but when it concerns these “traditional” conservatives (ie, the Moderate Party), this seems to be linked to a dimension of personal agency—being responsible and trustworthy—rather than to RWA. To eliminate the possibility that the differences originated from differences in the distribution of gender, education, age, and self-rated health over party affiliation, OLS regression analyses between a binary predictor of party affiliation (ie, Moderate Party versus the Liberal Party) and the stigma index items were performed. However, even in these adjusted models, the statistical differences remained (*P* < .05). However, the explanatory value of these models was low and ranged from R^2^ = 0.03 to R^2^ = 0.05.

Figure 2 shows the corresponding comparison between the supporters of the Moderate Party and the Sweden Democrats. In total, 5 of nine items displayed statistically significant differences at the .05 level: “If I had depression I would not tell anyone,” “People with depression are unpredictable,” “I would not hire a person if I knew they had been depressed,” “People with depression are dangerous,” and “I would not vote for a politician if I knew they had been depressed.” Thus, in the context of Sweden, the items most closely related to fear and uncertainty—being dangerous and unpredictable—displayed statistically significant differences between supporters of the Moderate Party and the Sweden Democrats (Figure 2) but not between supporters of the Moderate Party and the Liberal Party (Figure 1). When conducting OLS regression analyses adjusted for gender, education, age, and self-reported health, statistically significant differences (*P* < .05) remained for 2 items, namely, “If I had depression I would not tell anyone,” and “People with depression are dangerous.” The results suggest potential differences in the driving forces of stigmatizing attitudes even between different right-wing parties.

## Discussion

We found support for both hypotheses, ie, (1) that conservative ideology was associated with higher scores on stigmatizing attitudes toward depression even in a universal welfare state such as Sweden, and (2) that there were differences between different types of conservative supporters emphasizing either personal agency or responsibility and more populist right-wing supporters emphasizing predictability and fear. The collaboration between public health researchers and political scientists in the present project made it possible to comprehend the interdisciplinary dynamics beyond what would have been possible with only one of these disciplines. The observation of an association between conservative ideology and stigmatizing attitudes, even in a universal welfare state, contributes to previous knowledge of how the environment may play a part in relation to individual attitudes. That some items of the stigma scale showed clear “floor effects” may give some support to the dampening effect of universalistic welfare systems. However, the level of stigmatizing attitudes should be interpreted with caution because the self-recruitment of the present study sample might have led to overselection of people with a lower level of stigmatizing attitudes than in a random population sample (eg, a higher proportion with a high level of education). The type of welfare state may potentially shape attitudes and behavior toward marginalized groups in society by enabling or disabling shared notions of “outsiders.” This assumption was supported by an examination of media discourses on mental illness in 2 universal versus 2 selective welfare states: the media in the social democratic universal welfare states were most concerned with social inclusion and stigma, whereas media in the liberal selective welfare states emphasized danger and criminality, moving people with mental illness out of the mainstream.^25^ Beyond the media image, it is also possible that structural differences in, for example, health care and social security systems affect people’s attitudes based on the visibility of people with mental disorders in everyday life. One indication of this is that access to care seems to be much worse in nations with a selective welfare state than in nations with a universal welfare state.^26^ In parallel, Larsen,^27^ based on data from the World Value Survey, argues that political attitudes of individuals are highly influenced by institutional features such as the welfare state. In short, in a welfare state dominated by selective benefits and services, discussions on whether recipients are in need and whether recipients are to blame for their own situations are more pronounced and define the recipients as a special group distinguished from the “well-adjusted” majority. In contrast, in a welfare state dominated by universal benefits and services, discussions on whether recipients are in need and whether recipients are to blame are less pronounced, and recipients of welfare are defined as equal citizens.^27^ Following a similar line of reasoning, Pescosolido et al^2^ argued for a dynamic between the individual level, the community level, and the national level in the rise of stigmatizing attitudes. Their argument, concerning the role of the welfare state, was that more universal health care systems may instill norms of entitlement to health care in its citizens, which in turn may make citizens more likely to view health problems that are included in the national health care system as legitimate, and therefore, less stigmatized. However, a study by Aromaa et al^19^ on predictors of stigmatizing attitudes toward people with mental disorders in Finland indicates that any debate on the role of the welfare state should be about dampening effects rather than the elimination of stigmatizing attitudes. In Finland, which like Sweden is a comparatively comprehensive universal welfare state, 86% of the general population agreed that depression is a real medical disorder, but still a large number of respondents believed that people with depression are themselves responsible for their illness. However, the self-recruited sample in the present study limits the possibilities of generalizing the level of stigma to the whole population. Consequently, future cross-national studies with representative samples of general populations should investigate the association between level of stigma and type of welfare state further.

In a recent study, DeLuca et al^10^ observed how RWA predicted mental health stigma and they emphasized the need for further knowledge on why this association occurs. Our results may contribute to this and underpin the need to perform analyses in a multi-party system such as Sweden to reach generally valid conclusions. Party affiliation was used as a proxy for underlying ideology, and this measure is, admittedly, a rather weak indicator of the role of authoritarianism versus other forms of conservative views. Nevertheless, we observed significant differences between supporters of the Moderate Party and the Sweden Democrats. According to the view of voters in Sweden, the profile issues for the Moderate Party are jobs, taxes, and the economy, whereas the profile issues for the Sweden Democrats are immigration/refugees and law and order.^20^ In parallel, the current study showed that for supporters of the Moderate Party, stigmatizing attitudes seemed mainly to be driven by social distance manifested in agreement with statements such as “I would not vote for a politician if I knew they had been depressed” and “I would not hire a person if I knew they had been depressed.” But supporters of the Moderate Party also gave a comparatively high level of support to the statement “People with depression could snap out of it if they wanted,” which is in line with a worldview emphasizing personal agency and responsibility.^28^ Sweden Democrats seemed to stereotype based on fear and a vision of a homogeneous society. After controlling for gender, education, age, and self-reported health, we found a statistically significant difference between supporters of the Moderate Party and the Sweden Democrats for the statement “People with depression are dangerous,” with Sweden Democrats displaying the highest levels of support. This result is in line with the assumption that Sweden Democrats, but not supporters of the Moderate Party to the same extent, are guided by psychologic processes for managing fear and uncertainty. However, it is not clear whether this is generalizable for all RWA because a recent study in the United State did not find support for “dangerous world beliefs” as a mediator for this relationship.^10^ Still, our results underpin that forthcoming studies on the link between political ideology and stigmatizing attitudes toward depression should include multi-dimensional measures on political ideology. The distinction in previous research between liberalism and conservatism, and the strong focus on mechanisms related to psychologic processes for managing fear and uncertainty, is simply not sufficient to explain why ideology matters. Previous research has established that individual-level correlates such as female gender, higher education, younger age, and previous contact with individuals with mental illness are associated with less stigmatizing attitudes.^3,19^ The link to political ideology is less studied, and some scholars include political ideology without much reasoning on the causal mechanisms. For example, Thibodeau et al^4^ has written about “schematic knowledge” and predicted that liberals are likely to empathize with those suffering from depression and suggest medical treatment options. Other scholars link their reasoning on the role of political ideology to the relationship between conservatism and RWA.^3^ In this strand of research, genotype in interplay with environmental factors, including socialization and personal experiences of threatening events, is seen as a factor that may produce authoritarianism.^17,29^ The theoretical explanation for stigmatizing attitudes is thus that people with authoritarian preferences more often produce negative stereotypes of things that disturb their need for routine, predictability, and consistent behavior patterns. This makes them prone to view individuals with mental illness as dangerous.^3^ However, Feldman and Johnston^9^ criticized previous research on authoritarianism and stated (p. 346) that “the one-factor models of ideology provide a picture of determinants of ideology that is limited at best and, in several instances misleading.”

To summarize, depression is a leading cause of disability throughout the world,^11^ and stigmatizing attitudes may induce discrimination, delay in seeking help, and social isolation. From a public health perspective, it is therefore important to understand the mechanisms and drivers behind these attitudes. Based on the current results, there are reasons to believe that the type of welfare state people are living in affects the level of these attitudes and that a universal welfare state has a dampening effect.

That most participants, in all parties, disagree with statements such as “It is best to avoid people with depression” or “People with depression are dangerous” is good from the perspective of risk for inter-relational discrimination of individuals with depression. Based on the reciprocal relationship between public attitudes and formalized policy found in other areas of health-related policy,^30^ it is possible but not necessarily the case^31,32^ that stigmatizing attitudes toward people with depression influence voters’ attitudes to public expenditure, making them less supportive of generous social insurance programs and publicly funded treatment of individuals with depression. This should be investigated further. Somewhat more alarming is that a statement such as “I would not hire a person if I knew they had been depressed” received a comparatively high level of support. This kind of reasoning can lead to discrimination against individuals with depression on the labor market. Future studies should take the work context into consideration. From a public heath perspective, it is also relevant to note the relationship between self-stigma (ie, “If I had depression I would not tell anyone”) and political ideology; voters for the Sweden Democrats displayed the highest self-stigma, which may lead to delay in seeking help and social isolation. Future studies should investigate whether such a view also arises with seeking mental health care.

## Limitations

There are some methodological limitations that should be considered when interpreting the results of the present study. First, because of the self-recruited sample, generalizations of the average level of stigma toward depression should be done with caution. Second, because previous experience of depression might reduce the level of stigmatizing attitudes, analyses should preferably be adjusted for relevant variables. However, no such variables were attainable in the present study sample. On the other hand, no previous information indicates different levels of such experiences in relation to political ideology. Third, the observed floor effects on some items might indicate that the validity of the DSS might be uncertain, either due to the self-recruited sample (ie, a high proportion of highly educated) or due to an issue of “cultural validity” recently highlighted by Angermayer and Schomerus.^6^ Finally, the effect sizes observed were small and the results should be interpreted accordingly.

## Conclusion

In this study, we observed an association with political ideology: supporters for conservative parties scored higher on stigmatizing attitudes toward depression. The multi-party system in Sweden also provided an opportunity to observe differences in levels and potential drivers among supporters for different conservative parties. Stigmatizing attitudes in supporters for traditional conservative parties seemed to be driven by personal agency and responsibility, whereas supporters for the more populist right-wing party were stereotyped based on fear and social distance. The insight on these potentially different drivers contribute to the field and may also be relevant from a perspective of accuracy of future policy change or interventional strategies. However, the potential mechanism between stigmatizing attitudes toward depression and discriminating behavior toward people with this mental disorder needs more attention. The main conclusion is that we found stigmatizing attitudes toward depression in the Swedish context and that political ideology is part of that dynamic.

## Ethical issues

The study was performed in accordance with the Declaration of Helsinki. The Citizen Panel have obtained ethical approval from the Regional Ethical Review Board in Gothenburg, Sweden (Dnr 189-14).

## Competing interests

Authors declare that they have no competing interests.

## Authors’ contributions

The authors jointly designed the study and wrote the protocol. JM was responsible for the data collection and JL performed the data analyses. LW and JL drafted the manuscript. All authors discussed the results, contributed to, and approved on, the final manuscript before publication.

## Authors’ affiliations

^1^Department of Public Health and Community Medicine at Institute of Medicine, University of Gothenburg, Gothenburg, Sweden. ^2^Department of Political Science, University of Gothenburg, Gothenburg, Sweden.

## Key messages

**Implications for policy makers**

- That “drivers” of stigmatizing attitudes toward depression seem to differ with political ideology and party affiliation provides information for policy change and other societal interventions on this topic.
- Because of the relatively high support for the statement “I would not hire a person if I knew they had been depressed,” a particular focus on lowering stigmatizing attitudes on the labor market may buffer potential discrimination.

**Implications for public**

Stigmatizing attitudes of people with mental disorders, eg, they are a “danger to others” and declining “to live nearby someone with a mental health problem,” are a global challenge leading to discrimination, delayed care seeking, and social isolation. In order to change these attitudes, we need to understand the drivers. Previous research has indicated that political ideology could be one part of this puzzle, but more knowledge is needed to unravel the mechanisms behind the association between political ideology and stigmatizing attitudes, particularly in relation to the common mental disorder of depression. The present study shows how more conservative ideology provided more stigmatizing attitudes toward depression. The multi-party system in Sweden also gave us the opportunity to distinguish between different forms of conservatism where social distance and fear were more important drivers in populist right-wing voters compared with personal agency and responsibility among voters for the more traditional conservative party. This knowledge may guide future preventive actions toward stigma.
